# Supplementary material for: An archaellum filament composed of two alternating subunits
Source: Nat Commun. 2022 Feb 7;13:710. doi: 10.1038/s41467-022-28337-1 (PMC8821640; doi:10.1038/s41467-022-28337-1)
Supplement: Supplementary file 1 — Supplementary information [file 41467_2022_28337_MOESM1_ESM.pdf]

An archaellum filament composed of two alternating subunits

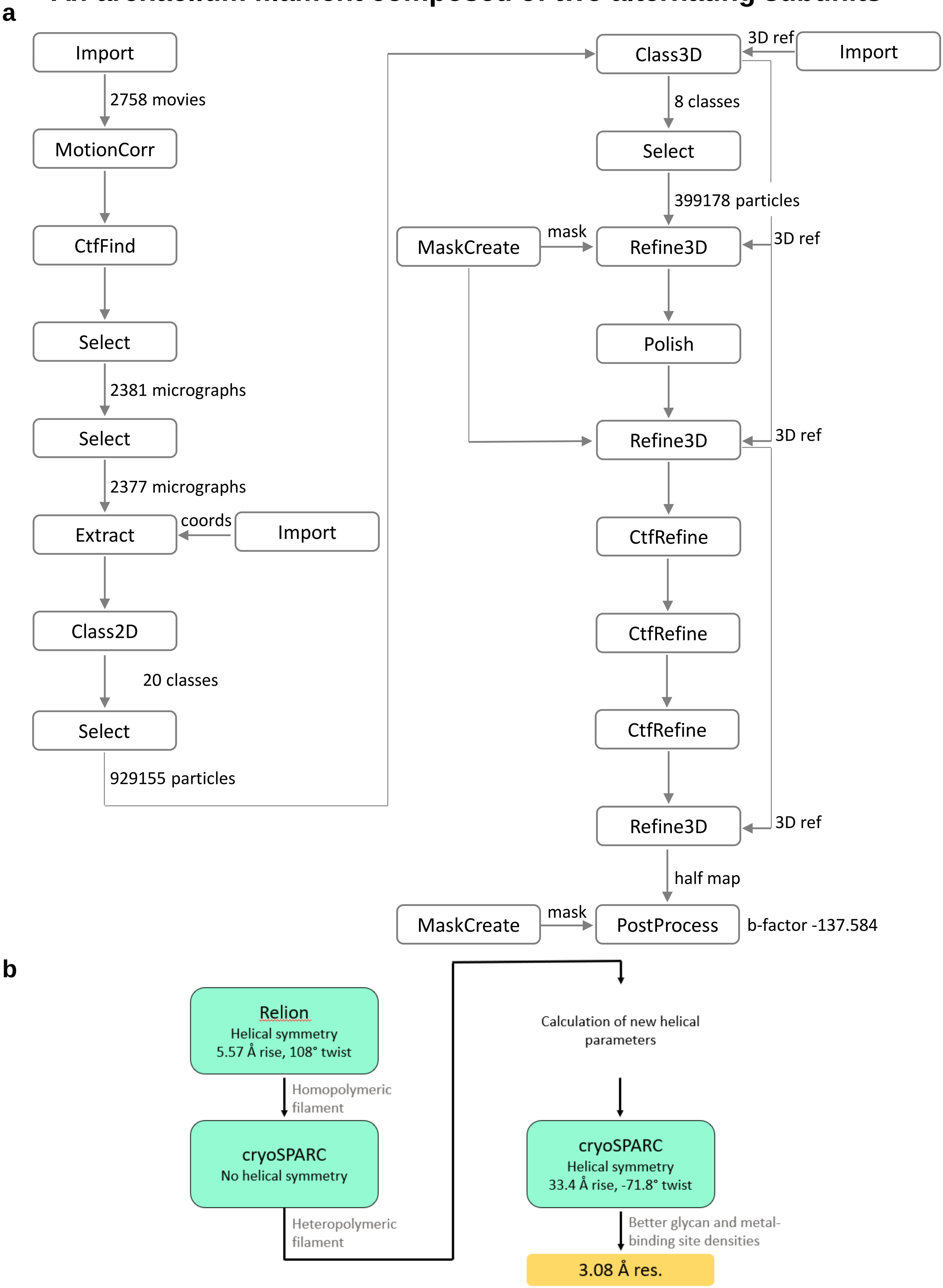

Supplementary fig. 1| Data processing flowcharts. Relion 3.1 (**a**) and cryoSPARC 3.1.0 (**b**) flowcharts.

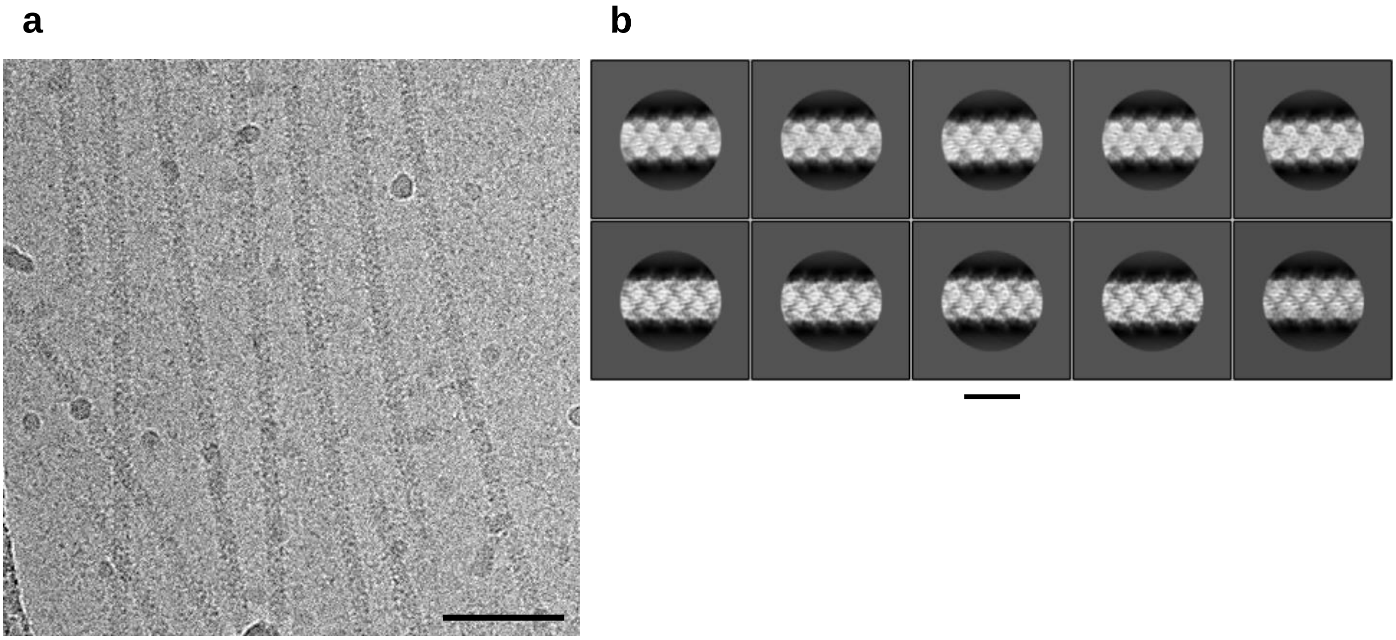

Supplementary fig. 2| Archaellum representative data. a, representative cryoEM micrograph (from a total of 2,759 micrographs) of *M. villosus* archaella. **b**, 2D classification examples of polished particles in Relion 3.1. Scale bar in (**a**), 50 nm; in (**b**), 10 nm.

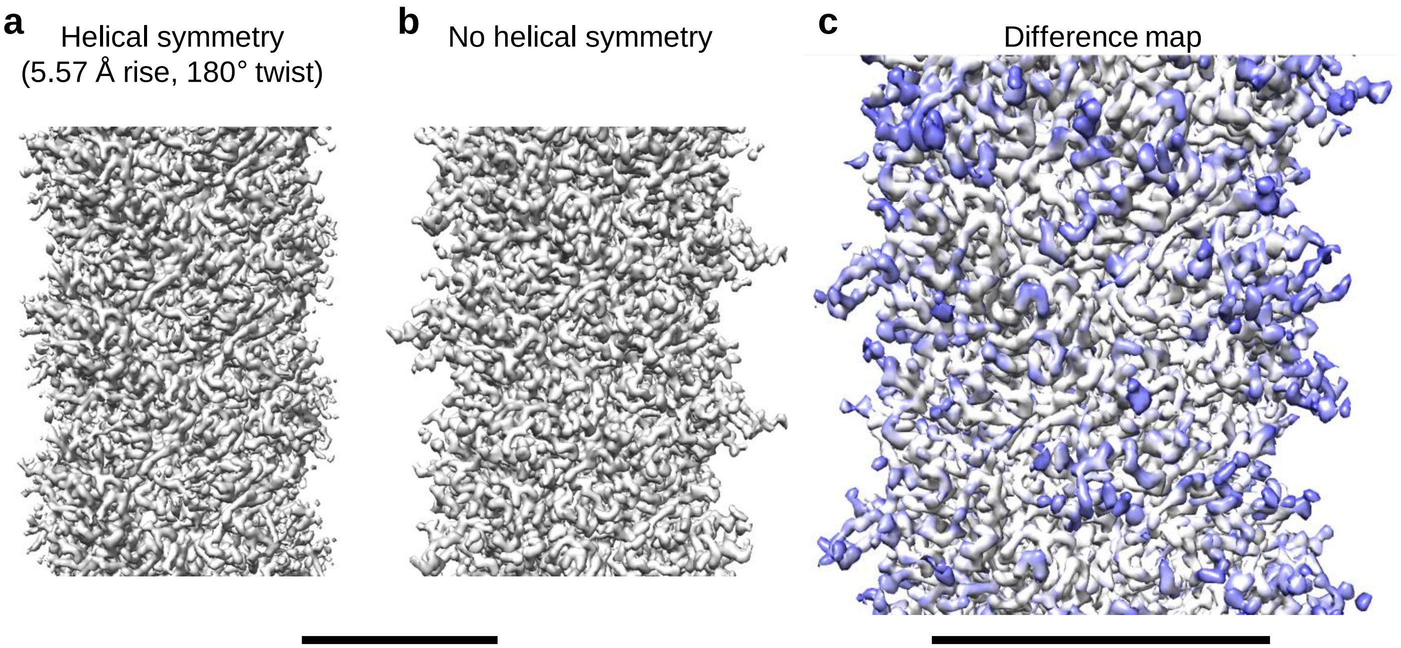

Supplementary fig. 3| Comparison between archaellum maps obtained with and without applying helical symmetry. CryoEM maps of the *M. villosus* archaellum filament obtained by applying helical symmetry (5.57 Å rise , 108° twist) (**a**), without helical symmetry (**b**) and difference map (**c**) between the maps shown in (**a**) and (**b**). The white areas of the map in (**c**) are those where the refined maps obtained with and without imposing helical symmetry agree. The blue areas are those better resolved without imposing helical symmetry and that were missing or fragmented in the map with helical symmetry. The areas that benefit from relaxing the helical symmetry (blue) are those occupied by glycans in ArlB1 and ArlB2 and by the "glycosylation loop" in ArlB2. Scale bar, 50 Å.

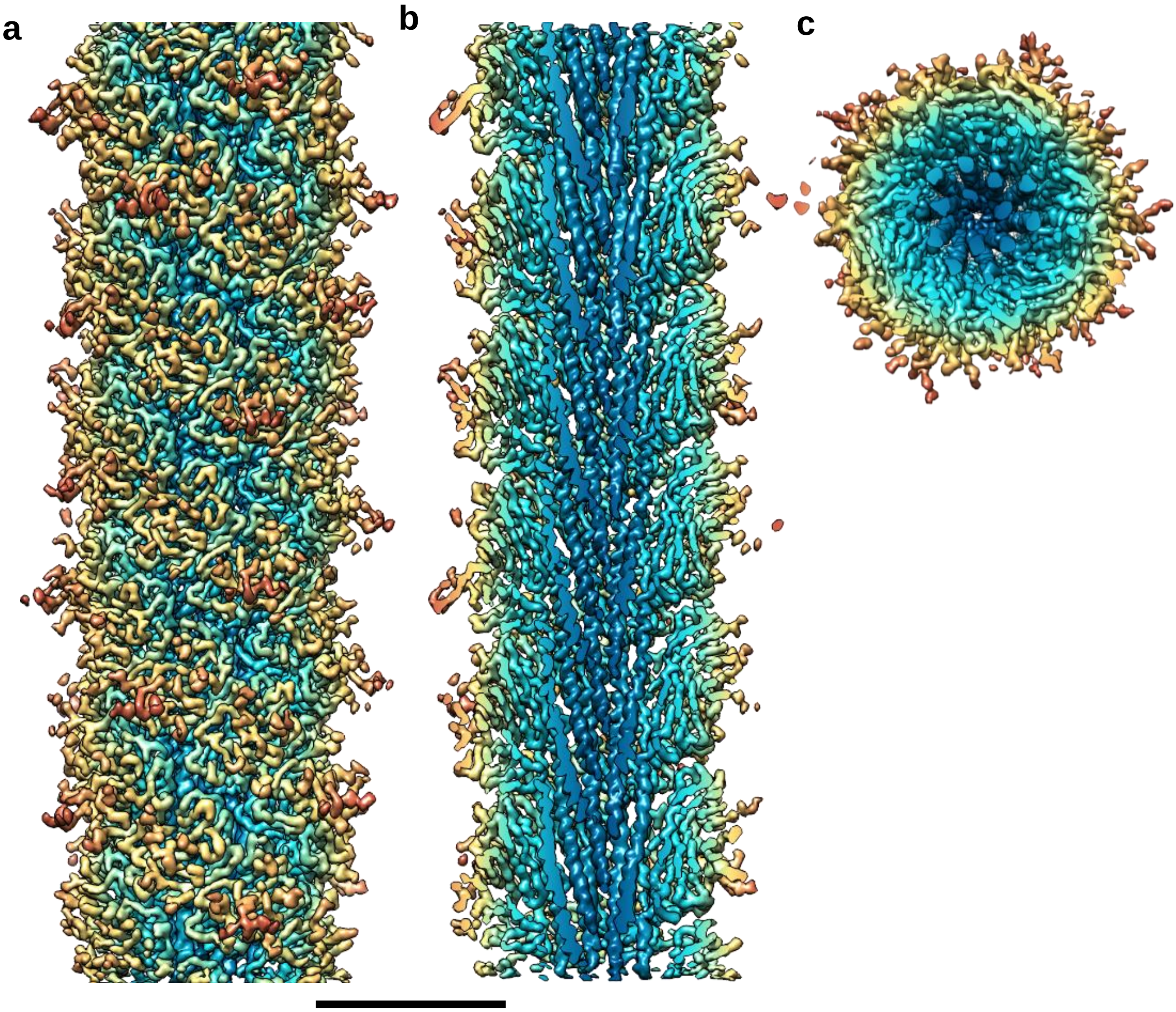

Supplementary fig. 4| *M. villosus* archaellum map. a, surface view; **b** and **c**, cross-sections parallel and perpendicular to the filament's long axis respectively. Colours range radially from the core (blue) to the periphery (orange) of the filament. Grooves on the surface in (**a**) follow the left-handed 3-start helix. Scale bar, 50 Å.

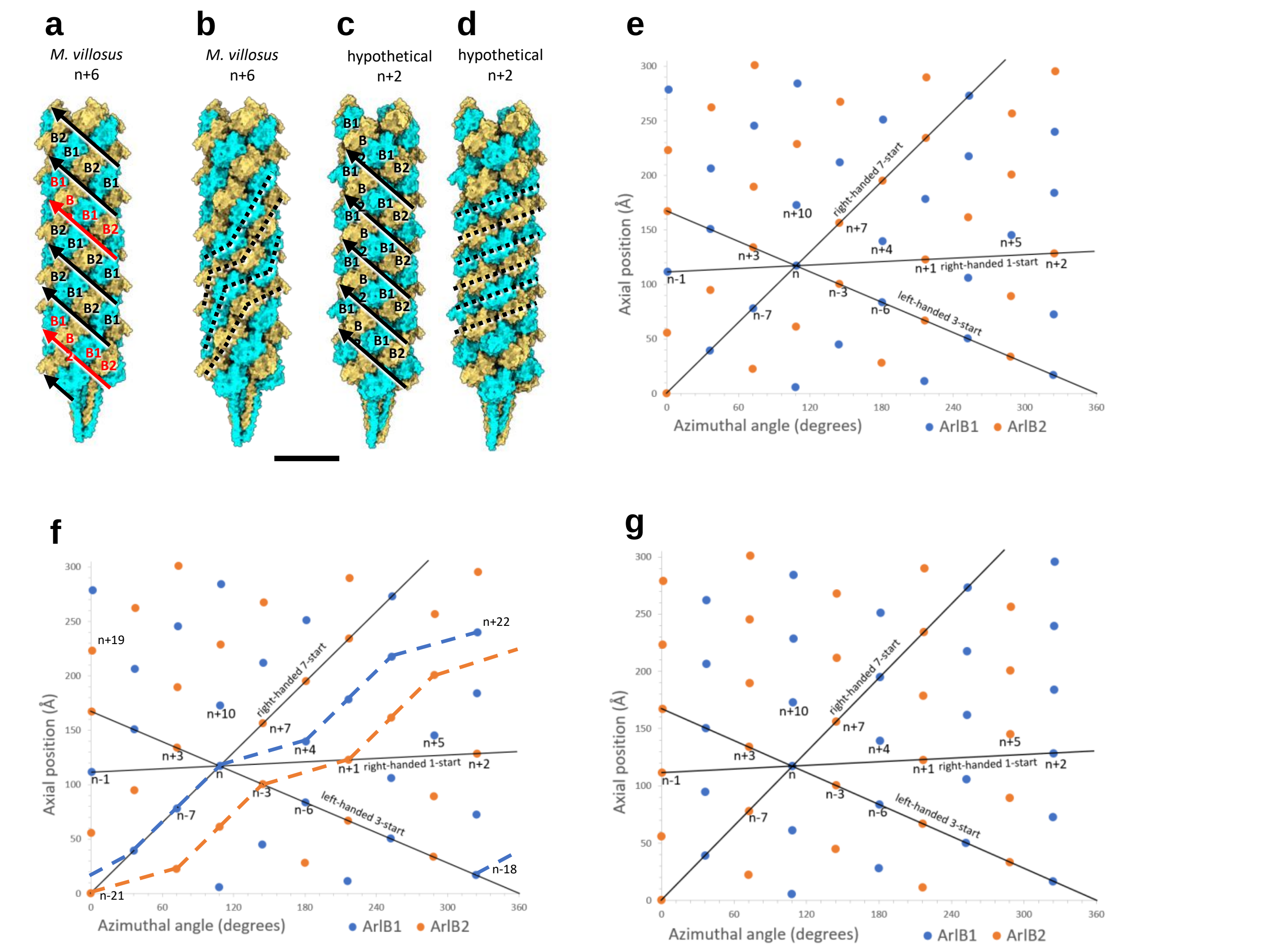

Supplementary fig. 5| Comparison between the *M. villosus* n+6 filament and a hypothetical n+2 filament. a-d, the heteropolymeric archaellum from *M. villosus* with n+6 symmetry (**a**, **b**) compared with a hypothetical filament with n+2 symmetry (**c**, **d**). **a**, in the *M. villosus* filament every third 3-start strand (red) is out of register with respect to the other 3-start helices (black arrows). **b**, subunits of the same type (ArlB1 or ArlB2 only) follow right-handed pseudo strands with broken symmetry. **c**, in a hypothetical n+2 filament, all 3-start strands are perfectly in register. The filament is isotropic and consists of either perfectly alternating or homopolymeric component strands. **d**, the two component subunits form true homopolymeric right-handed 4-start strands. Scale bar, 50 Å.

e, f, Helical net representations of the arrangement of protein monomers in the *M. villosus* heteropolymeric archaellum, as shown in (**a**) and (**b**). The view is from the outside onto the unrolled surface. Similar to homopolymeric archaella, the right-handed one-start helix with helical parameters (108°, 5.6 Å) passes through each protein monomer, the latter are labelled along this helix (e.g., n−1, n, n+1, etc.). The head domains make significant contacts along the right-handed 7-start helical strands (n+7) and the left-handed 3-start strands (n+3), whereas the tail domains also make contacts along the 1-start strand and a 4-start strand. The protein monomers of each type are shown as blue and orange dots, respectively. **e**, the diagram indicates a helical symmetry n+6. The ArlB1-2 subunits alternate along the left-handed 3-start strand, however, they have more complex order along the 1-start or 7-start strands. **f**, nearest monomers of the same type are shown as dashed lines (ArlB1 blue, ArlB2 orange). Eight molecules (including the first and the last near equivalent ones) are related by either n+7 or n+4 contacts and represent a full homopolymeric turn around the filament axis. **g**, a hypothetical heteropolymeric arrangement without screw axis asymmetry, as shown in (**c**) and (**d**). Here, the 3-start helix is built up from alternating monomers of each type. It has n+2 helical symmetry, manifested by each 4-start and 10-start strand formed by the monomers of the same type, while monomers of different types alternate along 1-start and 7-start helices.

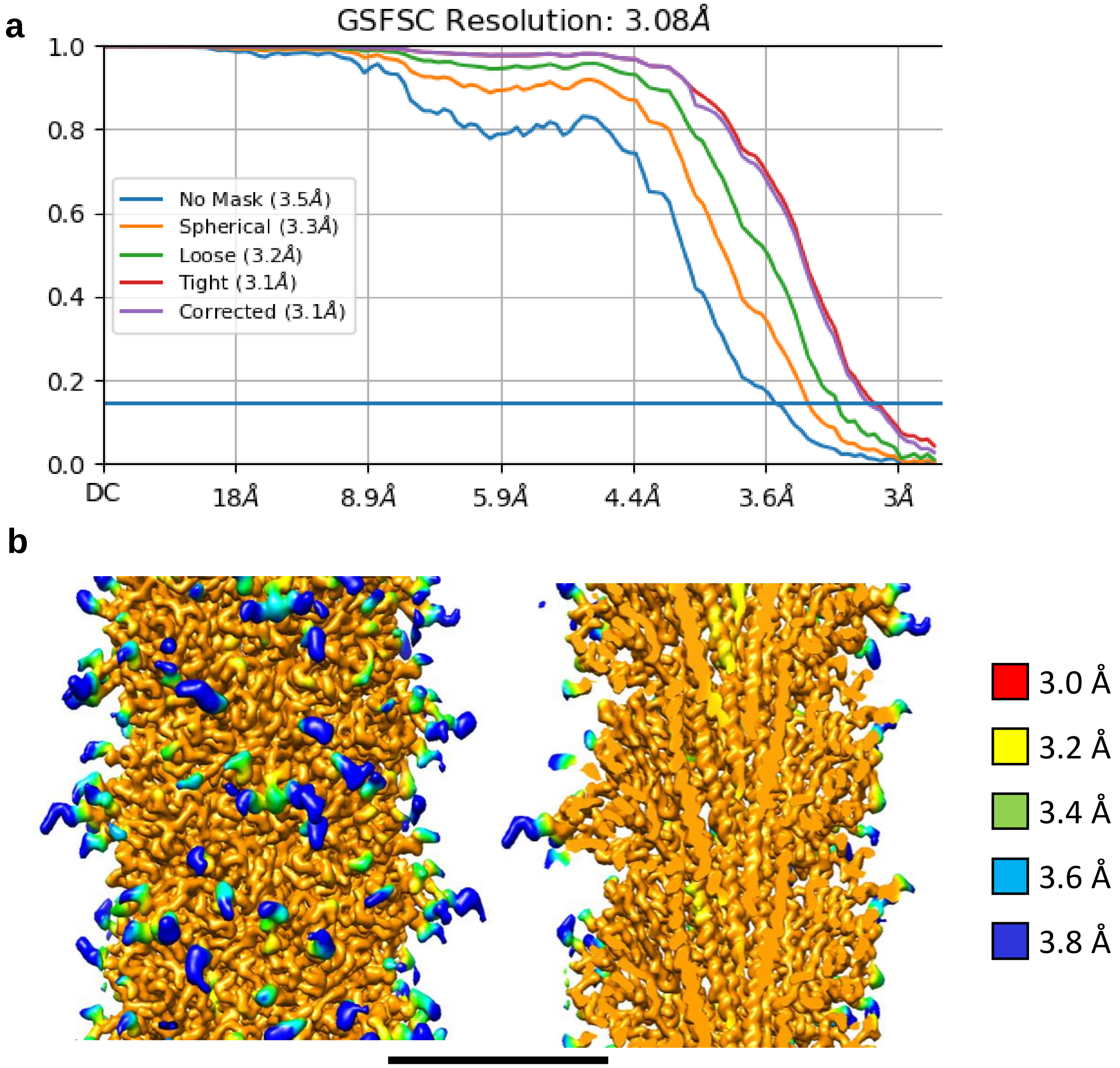

Supplementary fig. 6| Archaellum data quality. a, gold standard FSC and **b**, local resolution estimations for the archaellum map obtained from cryoSPARC 3.1.0 after helical refinement. Scale bar, 50 Å.

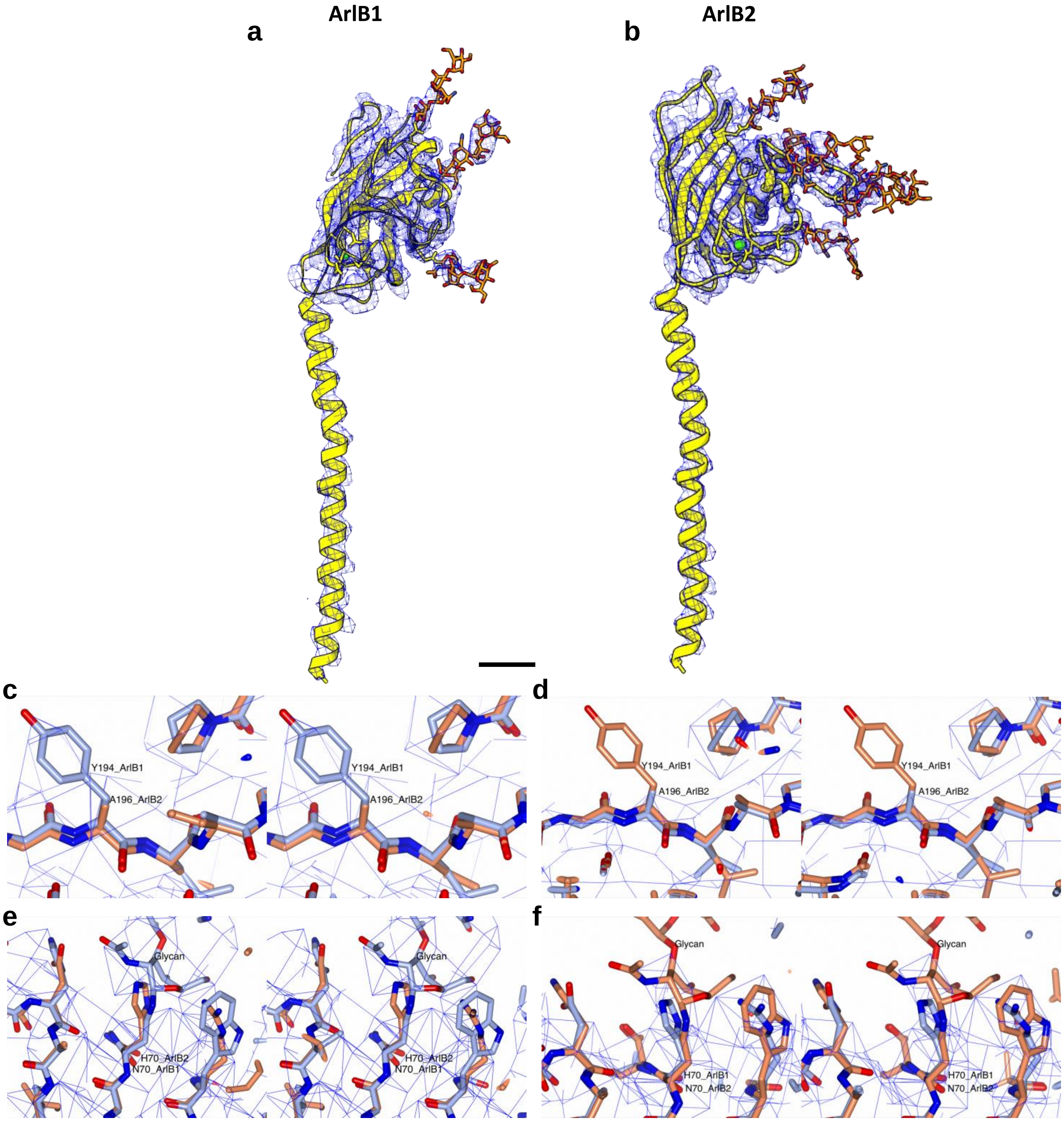

Supplementary fig. 7| Comparison between ArlB1 and ArlB2 cryoEM maps. a and **b,** atomic models of ArlB1 and ArlB2 (in ribbon representation, yellow; glycans in orange) respectively fitted onto the cryoEM maps (blue mesh). Scale bar, 10 Å. **c**-**f**, stereo views of large side chains (**c** and **d**) and glycosylation sites (**e** and **f**) in which the atomic models of ArlB1 and ArlB2 unambiguously fit the cryoEM density maps. The correctly fitting atomic model is shown in light blue, the incorrectly fitting atomic model is in copper, the cryoEM map is in blue mesh.

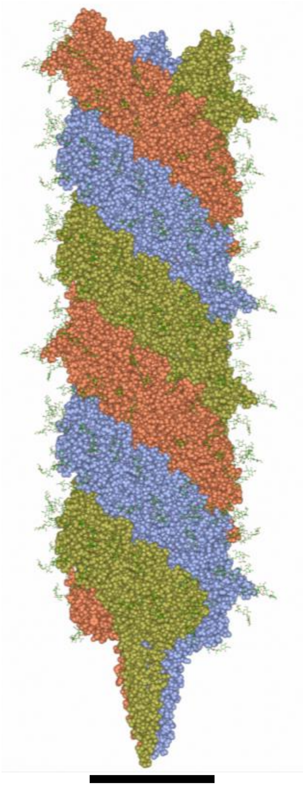

Supplementary fig. 8| Atomic model of the *M. villosus* archaellum showing the 3-start helical strands. Single 3-start strands are coloured in ice blue, gold and coral. Each 3-start strand is a heteropolymeric thread consisting of alternating ArlB1 and ArlB2. Protein atoms are displayed as spheres. Sugar moieties are shown as stick models. Scale bar, 50 Å.

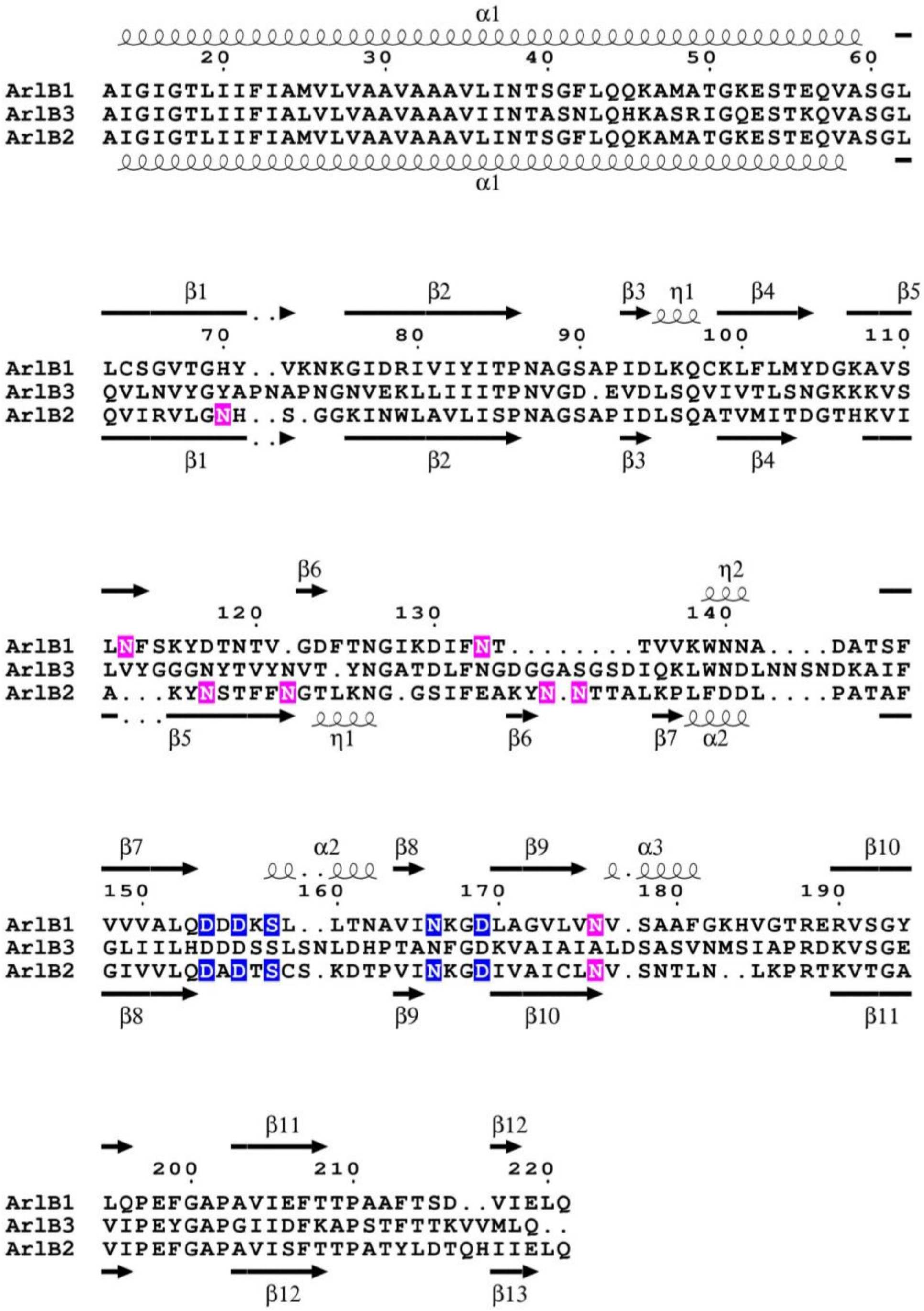

Supplementary fig. 9| Sequence alignment of *M. villosus* ArlB1, ArlB2, ArlB3 and secondary structure assignment of ArlB1 and ArlB2 archaellins. Secondary structure is indicated above and below the sequences as springs ("α" for α-helices and "η" for 3_{10}-helices) or arrows ("β" for β-strands). N-glycosylated residues are highlighted in pink and metal ion binding residues are highlighted in blue.

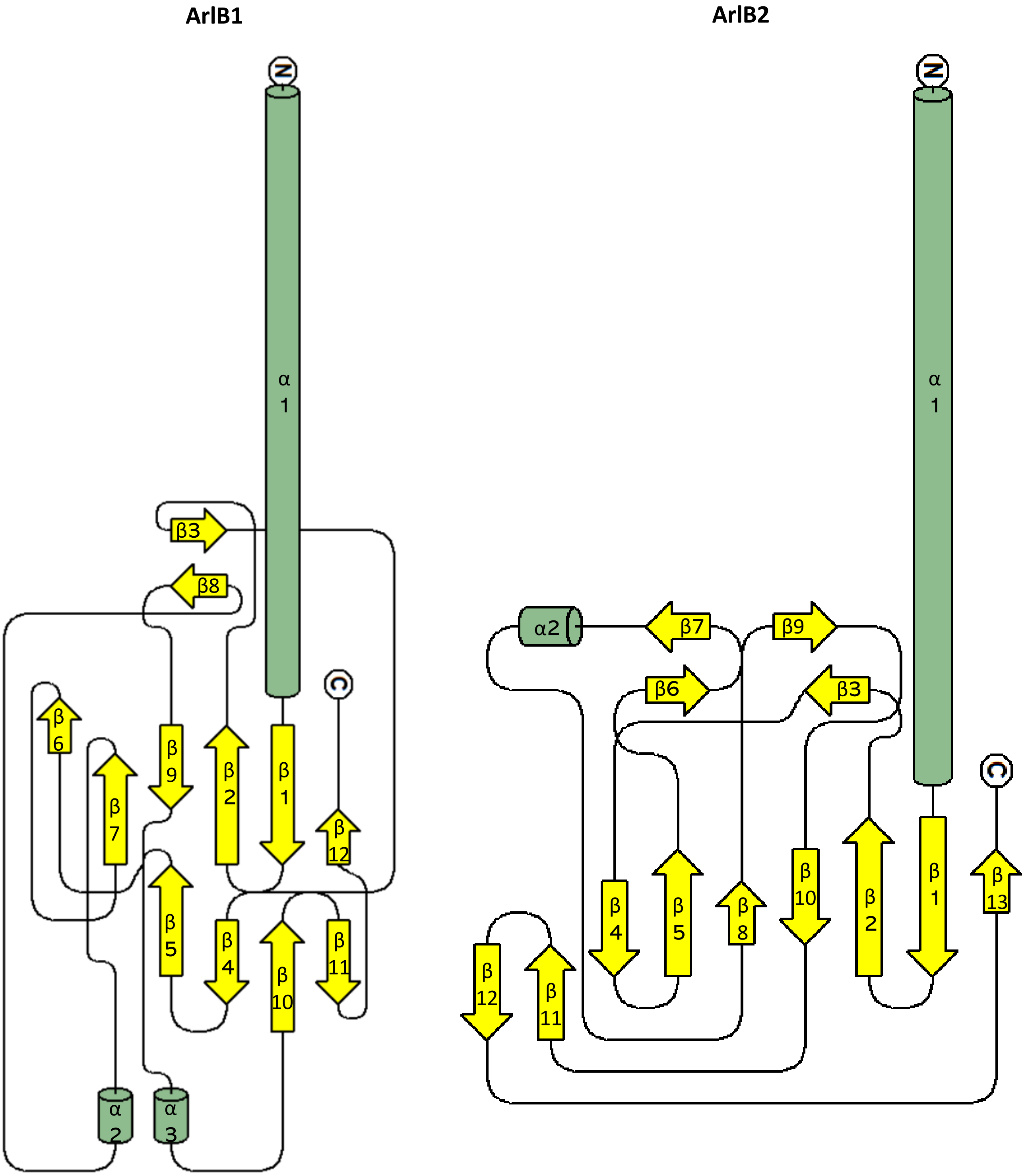

Supplementary fig. 10| Topology diagrams of ArlB1 and ArlB2.

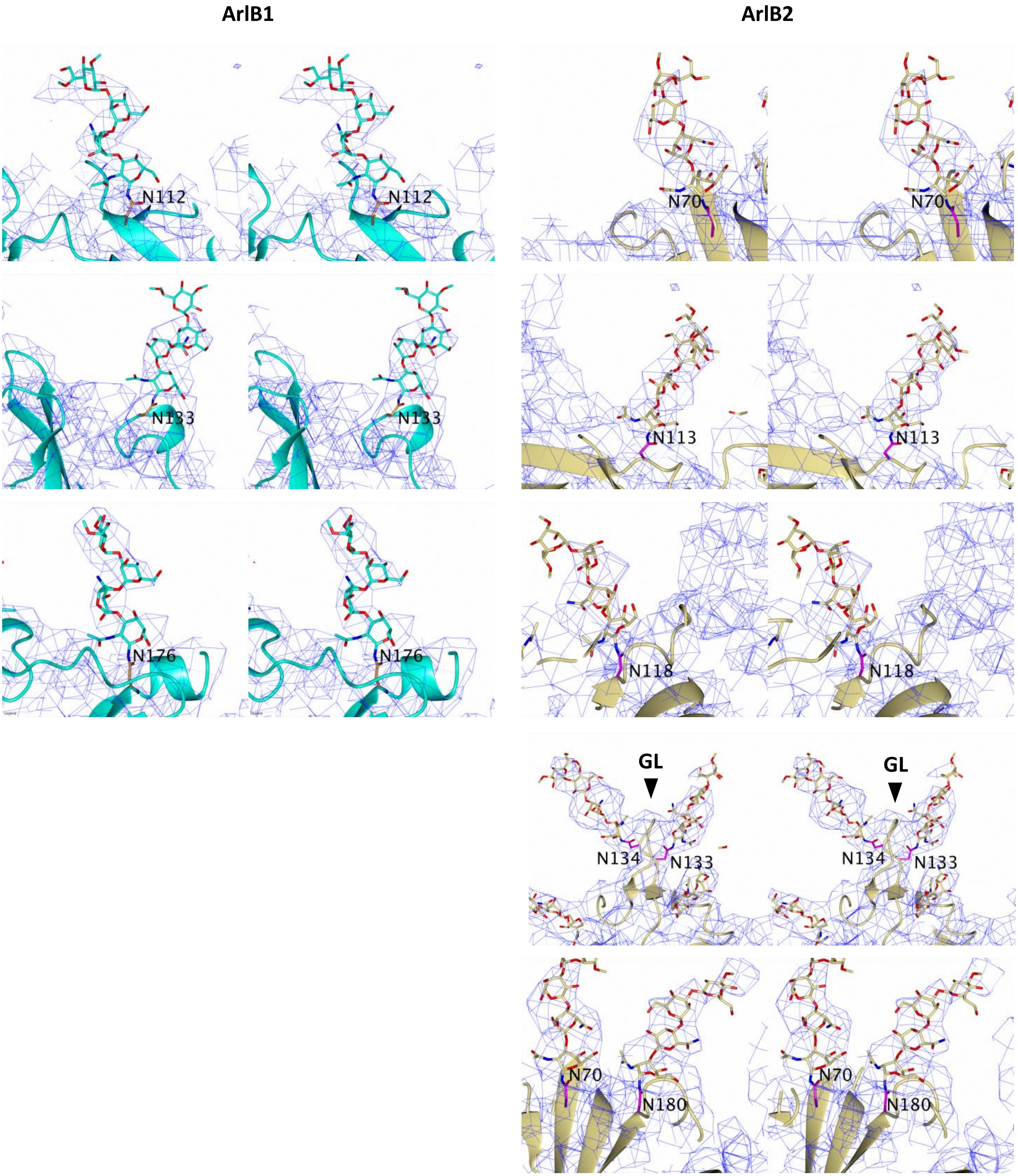

Supplementary fig. 11| Stereo diagrams showing the glycosylation sites in ArlB1 and ArlB2. ArlB1 and ArlB2 are shown as protein ribbon in cyan and sand respectively; the electron density maps are in blue mesh. Carbohydrates and side chains of glycosylated Asn residues are shown as stick models. Asn residues are additionally highlighted by differing carbon colours. GL, glycosylation loop.

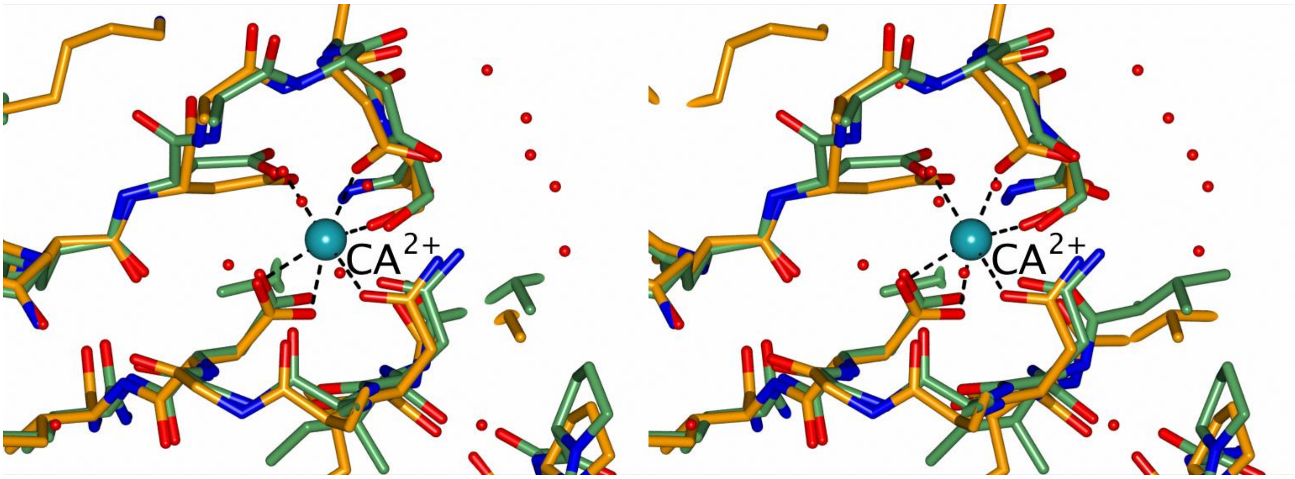

Supplementary fig. 12| Stereogram of the superposition of metal sites between *M. villosus* ArlB2 and *M. jannaschii* ArlB1. The two structures are shown in stick representation, with *M. villosus* ArlB2 in orange and *M. jannaschii* ArlB1 in green (X-ray structure, PDB ID: 5YA6, http://10.2210/pdb5ya6/pdb) showing a high degree of structural conservation.

a **b**

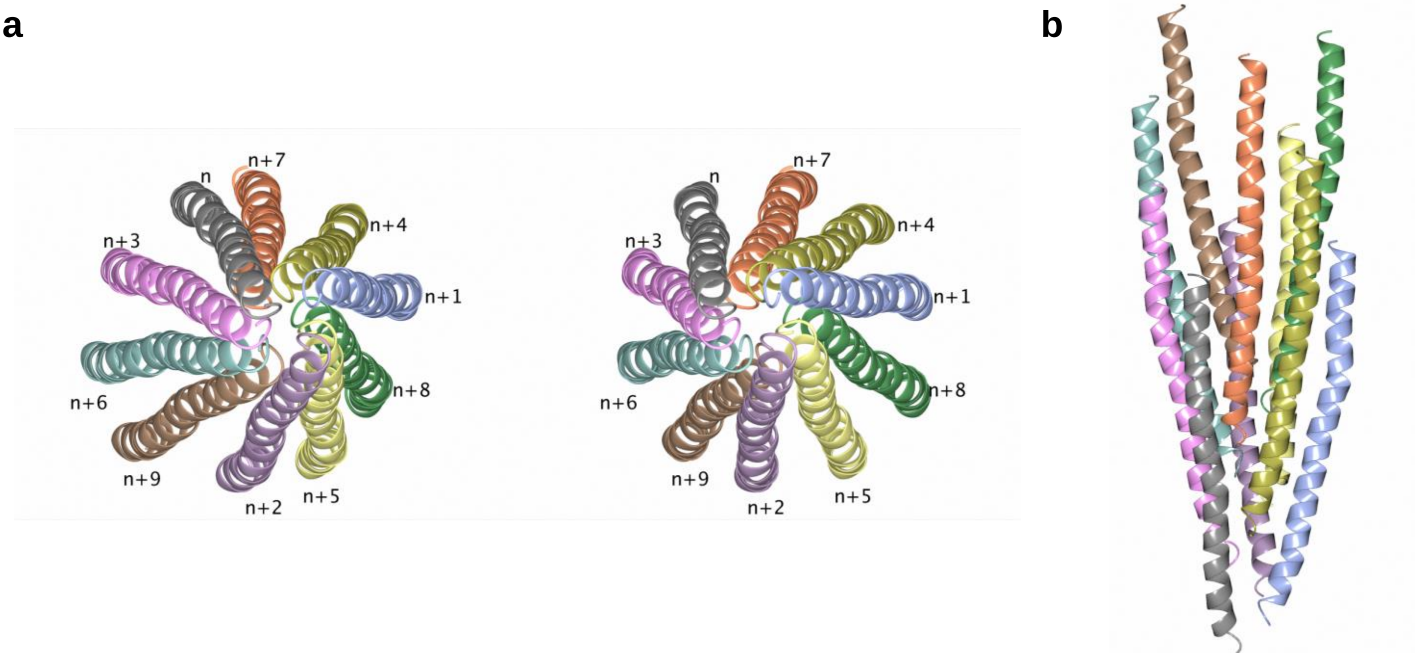

Supplementary fig. 13| Interactions between the tail helices in the archaellum filament. a, a stereo diagram showing ten tail domain helices viewed along the filament axis with each helix coloured differently and numbered in relation to the first one (n). **b**, helices from (a) viewed along the axis perpendicular to the filament.

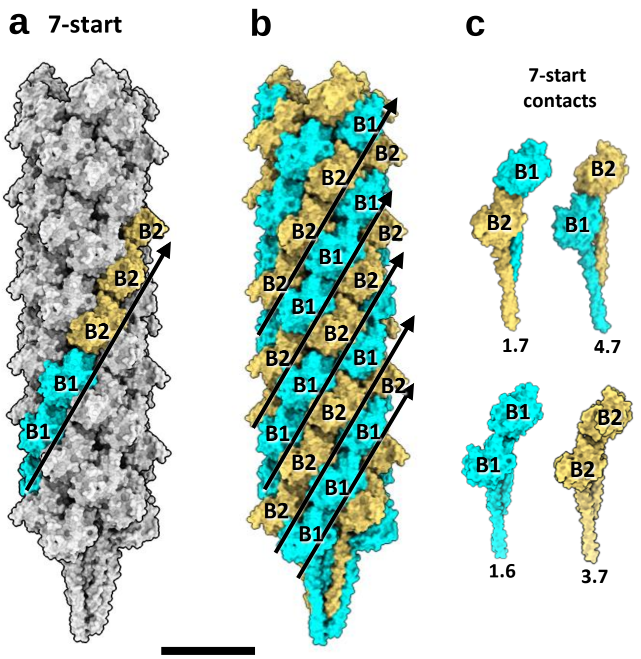

Supplementary fig. 14| Archaellum map with highlighted interactions along the 7-start direction. a, one right-handed 7-start strand and **b**, all 7-start strands are highlighted (direction of the 7-start strands indicated with black arrows). **c**, four types of contacts with various dissociation energies occur within the 7-start strands. The dissociation energies (ΔG_{diss} calculated by PISA) for each interaction are indicated below each dimer (unit: kcal/mol). Scale bar, 50 Å.

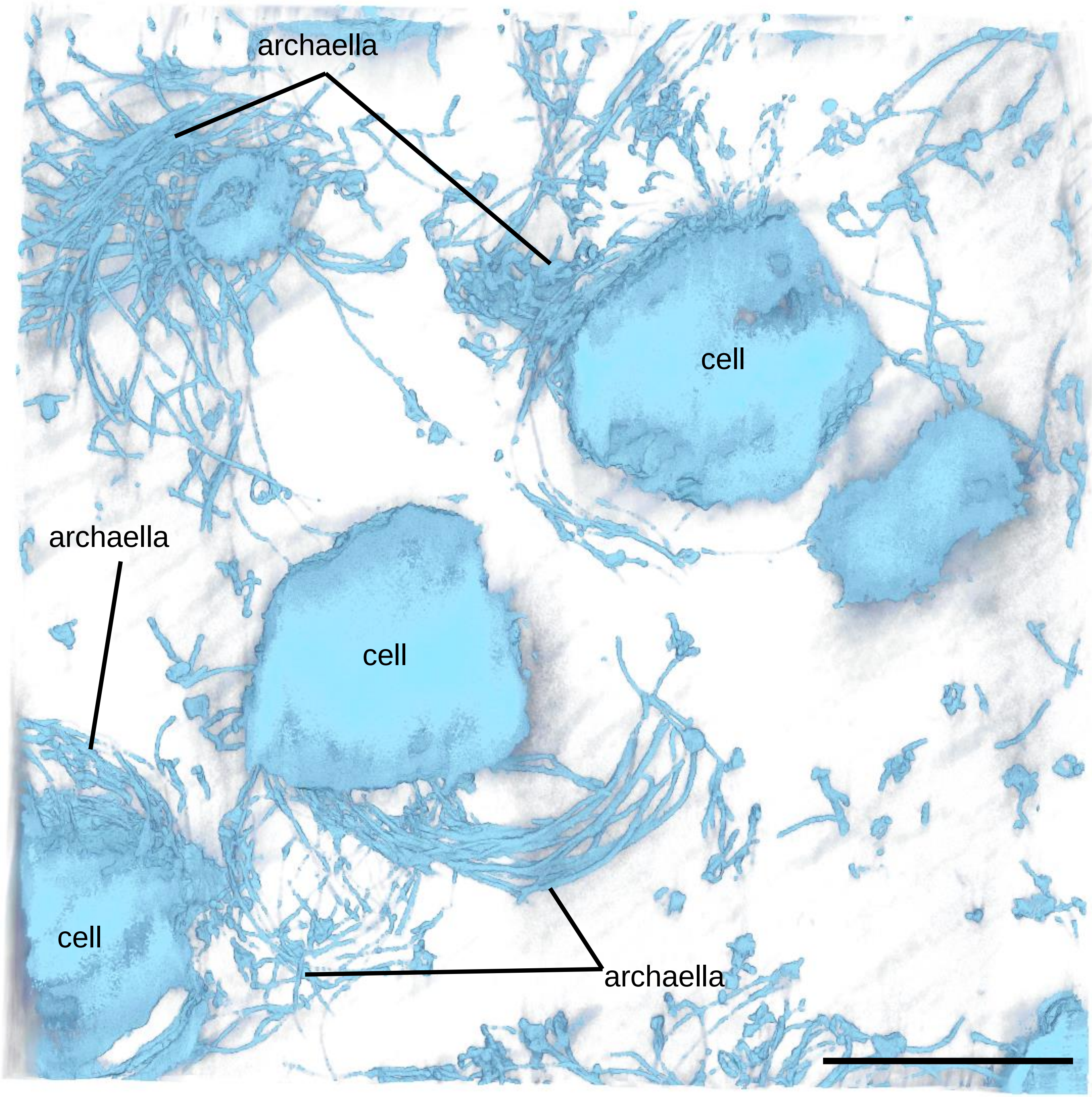

Supplementary fig. 15| STEM tomogram of freeze-substituted *M. villosus* cells. 3D representation was generated using solid representation in UCSF Chimera-X. Scale bar, 1 μm.

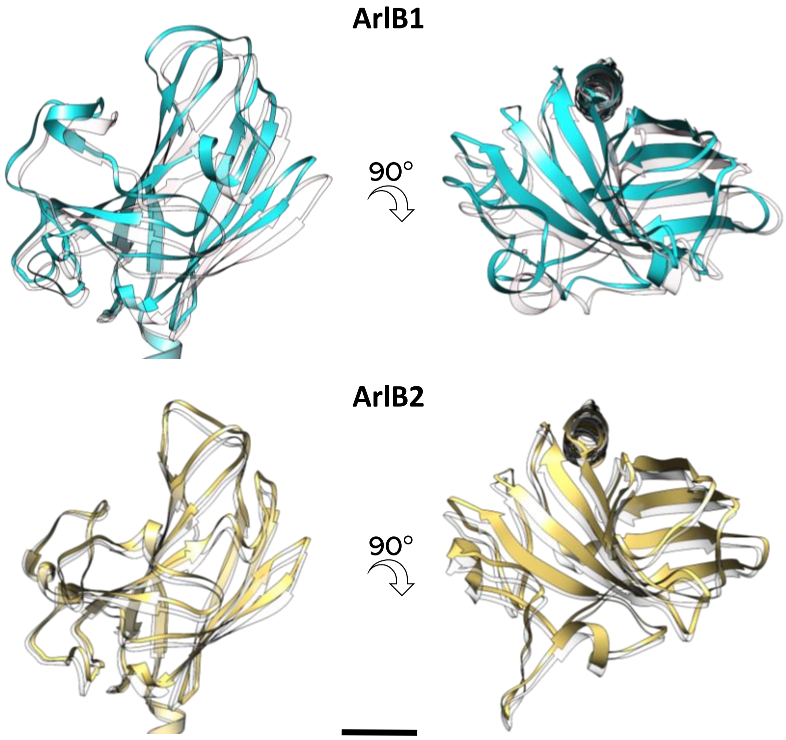

Supplementary fig. 16| Atomic models of one ArlB1 and one ArlB2 head domain showing their displacement during filament motion. The tail domains were aligned using *Coot*. The solid cyan (ArlB1)/sand (ArlB2) and transparent white models were fitted into the frame0 and frame19 maps of the cryoSPARC 3D variability analysis (3DVA) respectively. The models highlight a displacement diagonal to the filament axis. Scale bar, 10 Å.

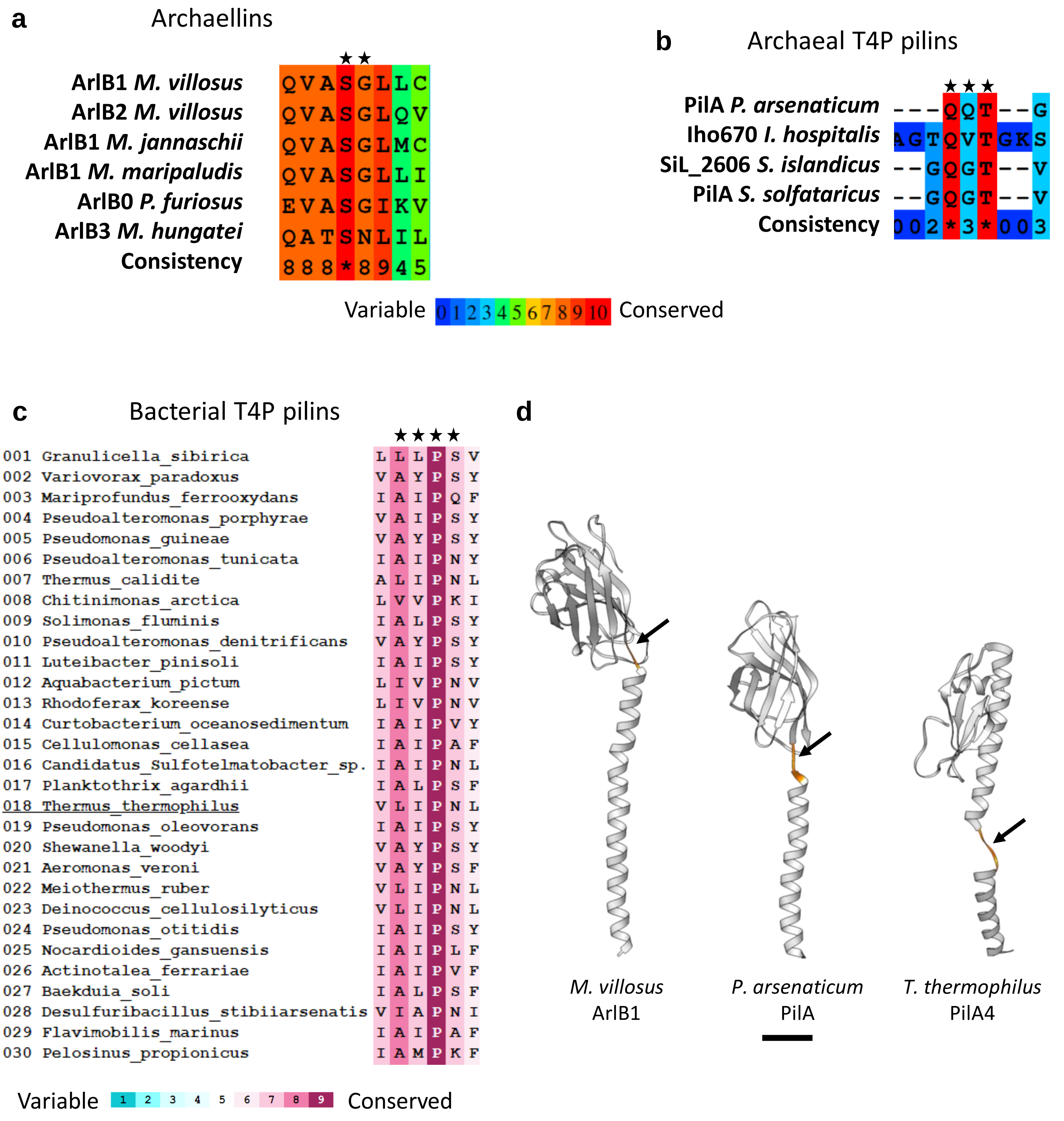

Supplementary fig. 17| Multisequence alignment of the hinge region of archaellins, archaeal T4P pilins, and bacteria T4P pilins. For archaellins (**a**) and archaeal pilins (**b**) the multi sequence alignment was performed in Praline and only species with known atomic structure were selected. The hinge regions are starred. For the bacterial T4P pilins (**c**) the ConSurf server was used with the UNIREF90 database and *Thermus thermophilus* PilA4 (PDB ID: 6XXD, https://10.2210/pdb6xxd/pdb) as template. The starred amino acids in (**c**) highlight the *T. thermophilus* PilA4 hinge region. In **d**, a comparison between atomic models of archaellins (*M. villosus,* ArlB1), archaeal T4 pilins (*P. arsenaticum,* PilA, PDB ID: 6W8U, https://10.2210/pdb6w8u/pdb6XXD), and bacterial T4 pilins (*T. thermophilus*, PilA4, PDB ID: 6XXD) shows the conserved hinge and loop regions (orange and arrows). Scale bar, 10 Å.

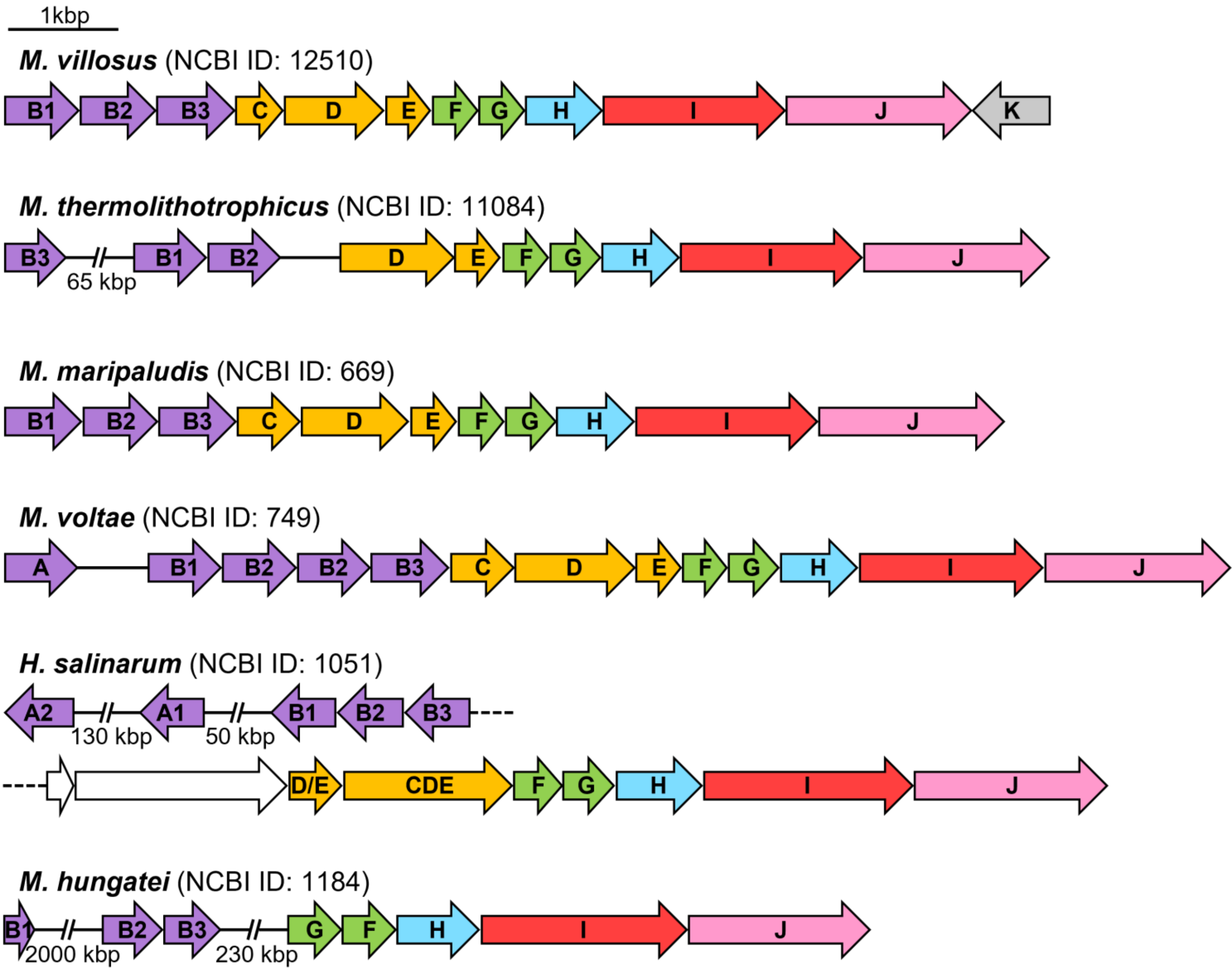

Supplementary fig. 18| Archaellum operons of archaea in which a hook at the base of the archaellum filament has been suggested. Purple: archaellins ArlA and B; orange: ring-forming proteins ArlC, D and E; green: periplasmic stator proteins ArlF and G; light blue: regulator protein ArlH; red: ATPase; pink: platform protein ArlJ; grey: prepilin peptidase ArlK; white: hypothetical protein.

a

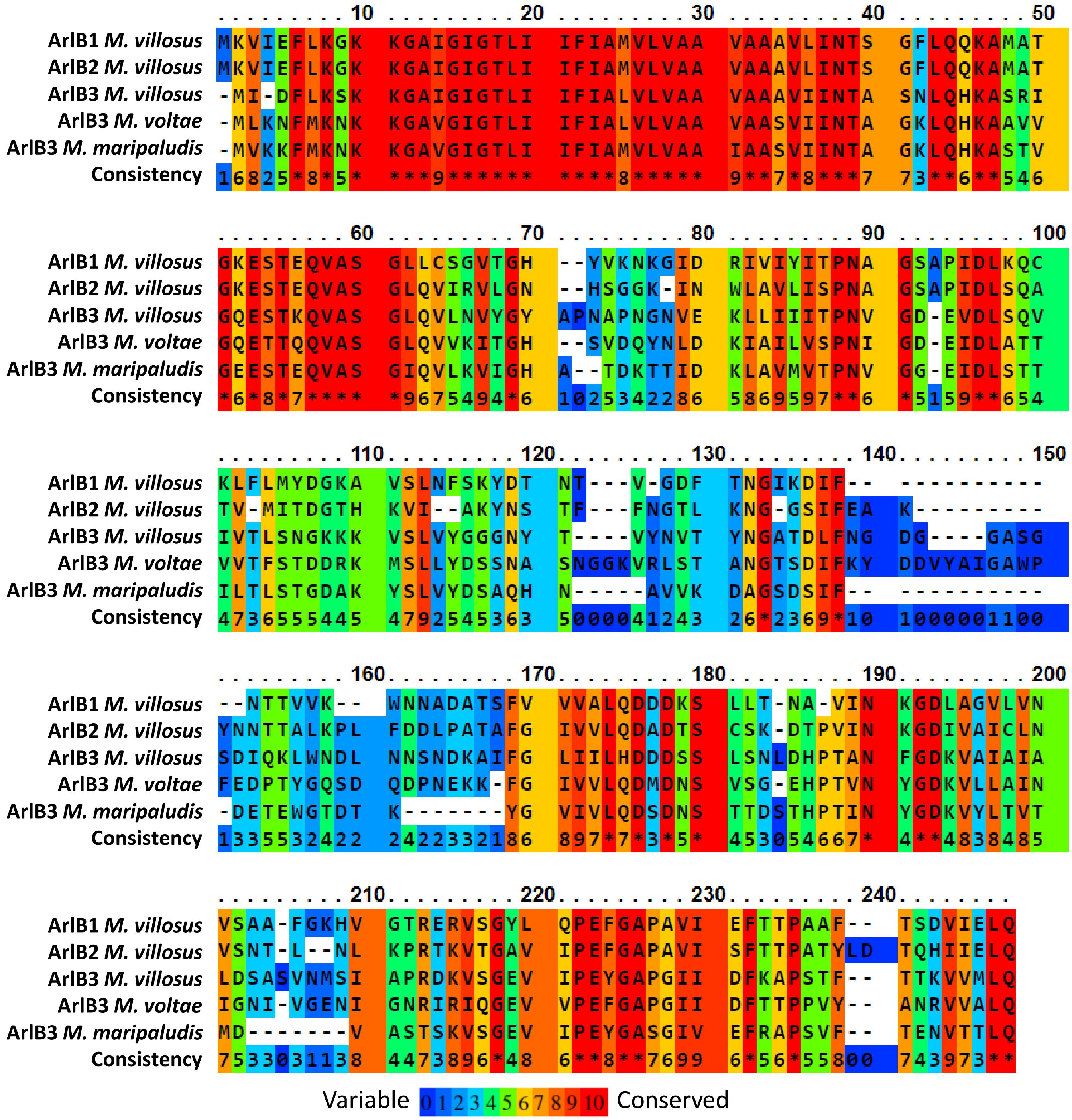

b

	M. villosus ArlB1	*M. villosus* ArlB2	*M. villosus* ArlB3	
M. voltae ArlB3	97	97	98	sequence coverage (%)
	41.5	45.6	48.3	identity (%)
	68.6	69.7	78.5	similarity (%)
M. maripaludis ArlB3	99	99	98	sequence coverage (%)
	43.9	44.7	50.4	identity (%)
	70.6	71.9	75.4	similarity (%)

Supplementary fig. 19| Multisequence alignment and comparison between *M. villosus* archaellins and *M. voltae* and *M. maripaludis* putative hook-forming archaellins. a, multisequence alignment of *M. villosus* ArlB1, ArlB2, ArlB3, *M. voltae* ArlB3 and *M. maripaludis* ArlB3 performed with Praline. **b**, sequence comparison between *M. villosus* ArlB1, ArlB2, ArlB3 with *M. voltae* ArlB3 and *M. maripaludis* ArlB3 using Blastp.

**Data collection**	
Electron microscope	Titan Krios
Electron detector	Falcon III
Voltage (kV)	300
Defocus range (µm)	-2.3 to -1.1 in 0.3 increments
Pixel size ($Å^2$)	1.39
Total electron dose ($e^-/Å^2$)	37
Exposure time (s)	1
Number of fractions	39
Total movies	2,759
**3D reconstruction**	
Final particles	399,178 helical segments
Resolution (masked FSC=0.143), Å	3.08
B factor	-137.584
EMDB accession #	12875
**Model Refinement**	
PDB ID	7OFQ
Model resolution (FSC = 0.50/0.143), Å	3.35 / 3.06
Model refinement resolution, Å	3.08
Non-hydrogen atoms (overall/protein/metal/glycan)	80,691/70,194/45/10,452
Number of monomers (overall/ArlB1/ArlB2)	45/23/22; 1.5 full helical turns
**RMS deviations**	
Bond length (Å)	0.010
Bond angle (°)	2.33
**Ramachandran plot**	
Favoured (%)	95.00
Allowed (%)	4.99
Outliers (%)	0.01
**Validation**	
Rotamer outliers (%)	1.96
Molprobity score	1.72
Clash score	3.65

Supplementary table 1| Statistics of data collection, 3D reconstruction and validation.

Parameter/ Contact type	BSA[a] $Å^2$	Hydrogen bond	Salt bridge	dG_diss kcal/mol
n + 3 ArlB1 - 2	3,356	13	3	20.4
n + 3 ArlB2 – 1	3,414	8	2	16.2
n + 3 ArlB1 - 2 (head domains only)	1,529	12	2	0.9
n + 3 ArlB2 - 1 (head domains only)	1,686	7	1	-
n+7 ArlB1 - 1	1,794	0	4	1.6
n+7 ArlB1 - 2	1,663	0	2	1.7
n+7 ArlB2 - 1	1,795	0	5	4.7
n+7 ArlB2 - 2	1,731	0	5	3.7

[a] BSA - buried solvent accessible surface area

Supplementary table 2| Protein contacts in the heteropolymeric archaellum of *M. villosus.*
