# Supplementary material for: An archaellum filament composed of two alternating subunits
Source: Nat Commun. 2022 Feb 7;13:710. doi: 10.1038/s41467-022-28337-1 (PMC8821640; doi:10.1038/s41467-022-28337-1)
Supplement: Supplementary file 3 — Description of Additional Supplementary Files [file 41467_2022_28337_MOESM3_ESM.pdf]

Description of Additional Supplementary Files

File Name: Supplementary Movie 1
Description: CryoEM map of the M. villosus archaellum filament showing alternating subunits.

File Name: Supplementary Movie 2
Description: CryoEM map and atomic model of the M. villosus archaellum filament.

File Name: Supplementary Movie 3
Description: Morphing of the 20 cryoEM maps of the cryoSPARC 3D variability analysis showing the M. villosus archaellum flexibility.

File Name: Supplementary Movie 4
Description: Atomic model showing the flexibility of the M. villosus archaellum filament.

File Name: Supplementary Movie 5
Description: Atomic model of two opposite n+10 sets of protein monomers showing the flexibility of the tail domains.

File Name: Supplementary Movie 6
Description: Atomic model in backbone representation showing the flexibility of the head domain of ArlB1 along a full filament turn of a homopolymeric pseudo-strand.

File Name: Supplementary Movie 7
Description: Atomic model in backbone representation showing the flexibility of the head domain of ArlB2 along a full filament turn of a homopolymeric pseudo-strand.
