# Supplementary material for: An archaellum filament composed of two alternating subunits
Source: Nat Commun. 2022 Feb 7;13:710. doi: 10.1038/s41467-022-28337-1 (PMC8821640; doi:10.1038/s41467-022-28337-1)
Supplement: Supplementary file 11 — Reporting Summary [file 41467_2022_28337_MOESM11_ESM.pdf]

nature portfolio

Corresponding author(s): Dr. Bertram Daum

Last updated by author(s): Jan 11, 2022

Reporting Summary

Nature Portfolio wishes to improve the reproducibility of the work that we publish. This form provides structure for consistency and transparency in reporting. For further information on Nature Portfolio policies, see our Editorial Policies and the Editorial Policy Checklist.

Statistics

For all statistical analyses, confirm that the following items are present in the figure legend, table legend, main text, or Methods section.

n/a	Confirmed	
☒	☐	The exact sample size (*n*) for each experimental group/condition, given as a discrete number and unit of measurement
☒	☐	A statement on whether measurements were taken from distinct samples or whether the same sample was measured repeatedly
☒	☐	The statistical test(s) used AND whether they are one- or two-sided<br>*Only common tests should be described solely by name; describe more complex techniques in the Methods section.*
☒	☐	A description of all covariates tested
☒	☐	A description of any assumptions or corrections, such as tests of normality and adjustment for multiple comparisons
☒	☐	A full description of the statistical parameters including central tendency (e.g. means) or other basic estimates (e.g. regression coefficient) AND variation (e.g. standard deviation) or associated estimates of uncertainty (e.g. confidence intervals)
☒	☐	For null hypothesis testing, the test statistic (e.g. *F*, *t*, *r*) with confidence intervals, effect sizes, degrees of freedom and *P* value noted<br>*Give P values as exact values whenever suitable.*
☒	☐	For Bayesian analysis, information on the choice of priors and Markov chain Monte Carlo settings
☒	☐	For hierarchical and complex designs, identification of the appropriate level for tests and full reporting of outcomes
☒	☐	Estimates of effect sizes (e.g. Cohen's *d*, Pearson's *r*), indicating how they were calculated

Our web collection on statistics for biologists contains articles on many of the points above.

Software and code

Policy information about availability of computer code

Data collection	EPU 1.5 was used for data collection.
Data analysis	For data analysis we used the following software: Relion 3.1, cryoSPARC 3.1.0, MotionCorr 2, CTFFind 4, EMAN-2, DeepEMhancer, REFMAC5, Buccaneer, Isolde, JLIGAND, MOLREP, Phenix, EM-TOOLS, IMOD, ConSurf server, BLASTp, ESPript3, ARTEMIS, Praline server, UCSF Chimera 1.14, Chimera-X 1.3, Coot, CCP4mg, Pro-origami, TopDraw, PISA.

For manuscripts utilizing custom algorithms or software that are central to the research but not yet described in published literature, software must be made available to editors and reviewers. We strongly encourage code deposition in a community repository (e.g. GitHub). See the Nature Portfolio guidelines for submitting code & software for further information.

Data

Policy information about availability of data

All manuscripts must include a data availability statement. This statement should provide the following information, where applicable:

- Accession codes, unique identifiers, or web links for publicly available datasets
- A description of any restrictions on data availability
- For clinical datasets or third party data, please ensure that the statement adheres to our policy

The archaellum atomic coordinates and electron density map have been deposited in the Protein Data Bank (https://www.rcsb.org/) with accession number 7OFQ and in the EM DataResource (https://www.emdataresource.org/) with the accession number EMDB–12875. The raw data have been deposited in the EMPIARE database (https://www.ebi.ac.uk/empiar/) with accession number EMPIAR-10884. Other structural data used in this study are: P. furiosus ArlB0 (PDB ID: 5O4U, http:// 10.2210/pdb5o4u/pdb), M. jannaschii ArlB1 (5YA6, http:// 10.2210/pdb5ya6/pdb), T. thermophilus PilA4 (PDB ID: 6XXD, https://10.2210/pdb6xxd/pdb), P. arsenaticum PilA (PDB ID: 6W8U, https://10.2210/pdb6w8u/pdb).

Field-specific reporting

Please select the one below that is the best fit for your research. If you are not sure, read the appropriate sections before making your selection.

☒ Life sciences ☐ Behavioural & social sciences ☐ Ecological, evolutionary & environmental sciences

For a reference copy of the document with all sections, see nature.com/documents/nr-reporting-summary-flat.pdf

Life sciences study design

All studies must disclose on these points even when the disclosure is negative.

Sample size	The structure of the archaellum filament is an average obtained from 399,178 filaments sections from 2,759 micrographs.
Data exclusions	A part of the recorded dataset was randomly excluded during data analysis when the structural resolution limit was reached.
Replication	The experimental findings are the result of averaging methods, therefore no replication of the experiments is required.
Randomization	This is not relevant to the present study because it is not a statistical study.
Blinding	Blinding does not apply to this study because it is not a statistical study.

Reporting for specific materials, systems and methods

We require information from authors about some types of materials, experimental systems and methods used in many studies. Here, indicate whether each material, system or method listed is relevant to your study. If you are not sure if a list item applies to your research, read the appropriate section before selecting a response.

Materials & experimental systems

n/a	Involved in the study	
☒	☐	Antibodies
☒	☐	Eukaryotic cell lines
☒	☐	Palaeontology and archaeology
☒	☐	Animals and other organisms
☒	☐	Human research participants
☒	☐	Clinical data
☒	☐	Dual use research of concern

Methods

n/a	Involved in the study	
☒	☐	ChIP-seq
☒	☐	Flow cytometry
☒	☐	MRI-based neuroimaging
